# A rare case of coronary artery complication in a child with systemic juvenile idiopathic arthritis and macrophage activation syndrome: case report and literature review

**DOI:** 10.1186/s12969-023-00944-2

**Published:** 2024-01-02

**Authors:** Lian Zhang, Yanwen Wei, Ningjing Zeng, Lianyu Wang, Xinying Chen, Jinghua Yang, Xiaolan Xiao

**Affiliations:** 1 The Second Clinical Medical College, Guangzhou University of Chinese Medicine, Guangzhou, China; 2 Department of Pediatrics, The Second Affiliated Hospital of Guangzhou University of Chinese Medicine, Guangzhou, China; 3 Ying Lv’s Renowned Expert Inheritance Studio, Guangdong Provincial Hospital of Chinese Medicine, Guangzhou, China

**Keywords:** Systemic juvenile idiopathic arthritis (sJIA), Coronary artery involvement, Macrophage activation syndrome (MAS)

## Abstract

A rare case of coronary artery involvement in a child with Systemic Juvenile Idiopathic Arthritis (sJIA) complicated by Macrophage Activation Syndrome (MAS) is reported. The patient initially received an inaccurate diagnosis of Kawasaki Disease, sepsis, and mycoplasma infection and showed no improvement after Intravenous Immune Globulin (IVIG) treatment. Upon admission, symptoms included diffuse red rash, swelling of the limbs, lymph node enlargement, and hepatosplenomegaly. Post investigations, a diagnosis of sJIA and MAS was confirmed, and treatment involved a combination of hormones (methylprednisolone) and immunosuppressive drugs (methotrexate). The revealed widened coronary artery diameter was managed with a disease-specific treatment plan and prophylactic plus low-dose aspirin anti-coagulation therapy. Under this management, MAS was well controlled, and follow-ups showed normalization of the child’s coronary artery structure and function. This case and the associated literature review underscore the importance of early recognition, diagnosis, treatment, and long-term monitoring for children presenting with sJIA and MAS complicated by coronary artery involvement.

## Introduction

Juvenile idiopathic arthritis (JIA) is a diagnosis of exclusion that begins before the age of 16 years and includes all forms of chronic arthritis of unknown cause [1]. Systemic juvenile idiopathic arthritis (sJIA) is a rare subtype of JIA that can cause systemic inflammation involving multiple organs and tissues and even severe and fatal Macrophage Activation Syndrome (MAS) [2]. It is usually characterized by fever, rash, arthritis, lymph node enlargement, hepatosplenomegaly, and serositis [3], with rare coronary artery damage. We report a rare case of coronary artery involvement in a child with sJIA combined with MAS.

## Case presentation

A 10-month-old boy was admitted to the Guangdong Provincial Hospital of Traditional Chinese Medicine on 27 May 2022 with “recurrent rash and fever for 35 days”.He had been screened in other hospitals for respiratory viruses, cytomegalovirus, herpes simplex virus, rubella virus, fine virus B-19, chlamydia, fungi, tuberculosis, mycobacterium typhi, mycobacterium paratyphi, toxoplasmosis, and scrub typhus infections, and his blood samples showed leukocytosis (27 × 109/L) with neutrophilia. He was diagnosed with KD, sepsis, and mycoplasma infections. He was treated with intravenous immunoglobulin (IVIG,2 g/kg,7/5), aspirin, and various antibiotics (cefotaxime sodium, cefoperazone sulbactam, azithromycin, ceftazidime, meropenem in combination with linezolid). However, the fever and rash did not improve. He was his mother’s third child, born at term, and was not vaccinated against the new coronavirus but was vaccinated against Bacillus Calmette-Guerin, hepatitis B, polio, measles, and meningitis. He was breastfed until 6 months, after which he started complementary feeding. Growth and developmental milestones are age-appropriate, and there is no family history of genetic disorders other than alpha thalassemia.

On admission, the child was mentally tired, febrile, T: 38.9 °C, with slightly increased respiration and heart rate. A diffuse red rash on the head, face, limbs, and trunk faded on pressure and did not interfere with stroking. The rash resolved spontaneously without fever. The swollen limbs and several enlarged lymph nodes could be palpated bilaterally in the anterior neck, axillae, and groin, and the liver and spleen were palpable 5 cm below the ribs, confirmed by abdominal ultrasound. It showed that this supported the diagnosis of sJIA.

Further laboratory investigations revealed a significant decrease in leukocytes, hemoglobin, and platelets compared with the previous period, a significant increase in liver enzymes (AST: 1569 U/L, ALT: 521 U/L), a decrease in fibrinogen (FIB: 0.72 g/L), a progressive increase in ferritin and an increase in lipids and LDH, as shown in Table 1, suggesting the development of MAS comorbidities. The treatment regimen included intravenous methylprednisolone (2 mg/kg), hepatoprotective drugs (glutathione and glycyrrhetinic acid diamine) and oral MTX; subsequent bone marrow smear showed reactive phagocytosis of histiocytes, Ultrasonic Cardiogram (UCG) suggested bilateral coronary artery dilatation (max Z-value = 3. 52) and traces of pericardial effusion, and cytokine testing showed IFN-γ: 69.74 ng/L, IL-10: 20.21 ng/L, IL-6: 38.42 ng/L; NK cell activity 15.76% and soluble CD25: 14738 U/mL, consistent with macrophage activation.

**Table 1:** The findings of laboratory test

Date/Day of illness	2022/5/27(D1)	2022/5/28(D2)	2022/5/29(D3)	2022/5/30(D4)	2022/5/31(D5)	2022/6/01(D6)	2022/6/03(D8)	2022/6/06(D11)
ALT,U/L(9-50)	521	/	765	769	671	590	345	176
AST,U/L(15-40)	1569	/	1968	1446	737	331	116	77
GGT,U/L(10-60)	282	/	567	709	929	1042	897	399
TG,mmol/L(0.40-1.70)	2.73	/	4.15	/	4.74	/	/	4.81
FIB,g/L(2.00-4.00)	0.72	/	0.65	/	0.7	/	1.19	1.53
Fer,ng/ml(4.63-204.00)	>2000	/	>2000	>2000	/	/	>2000	4681.1
ProBNP,ng/L(0-125)	1832	/	2942	1275	580	/	/	/
WBCx10^9/L(5.0-12.0)	4.80	3.64	5.35	3.27	2.94	4.15	5.77	4.73
NEUT%(40.0-75.0)	53.0	44.8	29.8	30.8	17.9	45.6	14.3	10.0
Hb,g/L(120-160)	70	63	111	121	111	113	105	95
PLTx10^9/L(100-300)	155	73	100	118	101	103	105	63
CRP,mg/L(0.00-6.00)	71	/	38	24	13	7.9	2.19	0.58
PLTx10^9/L(100-300)	1.55	/	0.59	/	0.27	/	/	0.08
Other important findings	IgA:< 0.28	sCD25:14738	/	/	CD4/CD8:0.57	IgA:< 0.28	/	/
	IgM:0.37	/	NK:0.87; CTL:1.5	/		IgM:< 0.18		/

**Fig. 1:**
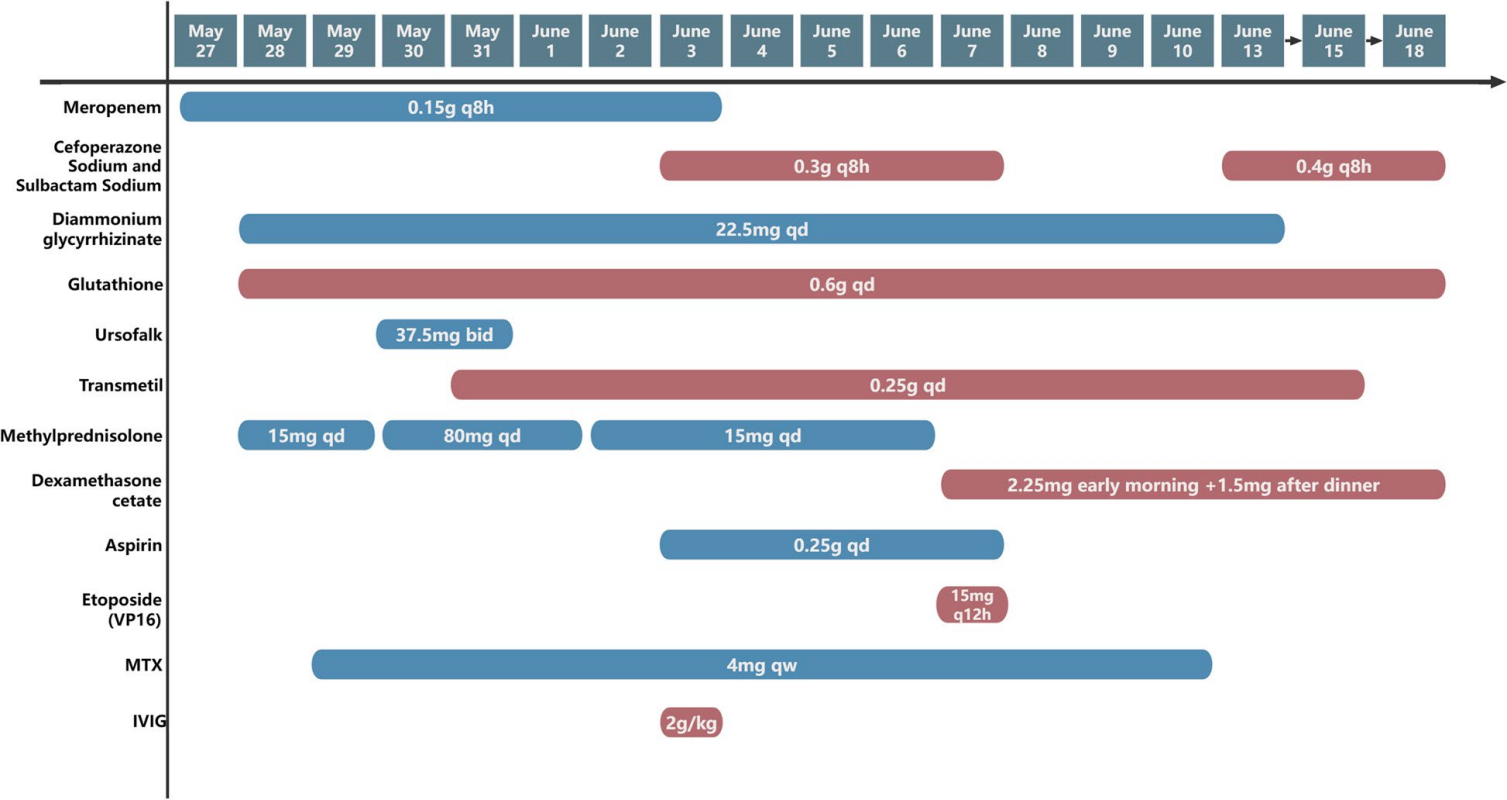
Treatment Process Diagram

Given the intense inflammatory storm, methylprednisolone shock therapy for 3 days (10 mg/kg). Although there was no evidence of definite efficacy of IVIG use in children with sJIA with coronary damage, treatment with aspirin and IVIG was continued, given the rapid progression of the disease. As the inflammatory storm was controlled, his body temperature returned to normal, the generalized rash resolved, limb swelling decreased, and liver and coagulation function improved compared with the previous period. The treatment regimen was changed to oral Dexamethasone acetate + etoposide (VP-16) and methotrexate by the HLH-1994 protocol and the recommendations of the out-of-hospital expert diagnosis and treatment. A further UCG suggested further coronary dilatation and anti-polymerization therapy with dipyridamole and aspirin was given. After stabilization, the child was discharged from the hospital. A follow-up UCG in the second month after discharge showed that the structure and function of the coronary arteries had returned to normal. The inflammation was under stable control, so dipyridamole and aspirin were discontinued, oral MTX was maintained, and the specific dosage and duration of the medication are shown in Fig. 1. The child is currently in good condition.

## Discussion

This is a case of delayed coronary artery injury presenting in a patient with sJIA and MAS. He presented with unexplained persistent fever for > 2 weeks (a requirement), resolving rash (1 major indicator), generalized lymph node enlargement and hepatosplenomegaly, plasmacytosis (pericardial effusion), and leukocytosis (3 minor indicators), which met the 2018 International Trials of Rheumatic Diseases in Childhood diagnostic criteria for sJIA. Elevated AST, ferritin, lipids, LDH, decreased fibrinogen, reactive phagocytosis of bone marrow histiocytes, and elevated CD25 all support the child’s comorbid MAS. What is more, elevated coronary Z-values measured on multiple UCG were consistent with small coronary aneurysms graded for KD coronary involvement. To our knowledge, this is a rare case of sJIA-associated coronary artery injury reported in mainland China.

JIA is one of the most common chronic rheumatic diseases in childhood, not only causing joint involvement and even joint deformity in children but also affecting other systems outside the joints and even leading to fatal complications such as MAS. Cardiovascular involvement due to JIA infection is a severe complication, including pericarditis, myocarditis, endocarditis, valvular heart disease, and heart failure, which can be fatal if left untreated [4]. Furthermore, the incidence of cardiovascular involvement in JIA is not low and is usually associated with a poor prognosis [5]. However, coronary artery disease appears to be rare in JIA and should be taken seriously.

JIA is the most common chronic rheumatic disease in children and a significant cause of short- and long-term disability. sJIA is a distinct subtype of JIA [6]. Cardiac lesions in sJIA are most commonly pericardial effusion; cardiomyopathy also occurs, and coronary artery lesions are rare [7]. Therefore, physicians usually rely on echocardiographic evidence of coronary artery abnormalities to differentiate between KD and sJIA; however, several recent studies have found that coronary artery disease is also present in children with sJIA. In the current literature, coronary involvement in children with sJIA is most commonly described as coronary artery dilatation (see Table 2 for details). A 6-year-old child with sJIA was misdiagnosed as having KD and MAS secondary to Epstein-Barr virus (EBV) infection. This child presented with dilatation of the left coronary artery and left anterior descending branch. The coronary artery damage was not repaired until 6 months after administration of methotrexate, steroids, cyclosporine, and prednisolone [8]. This suggests that our sJIA patients may need long-term monitoring and adherence to medications until the coronary damage is repaired. Myocardial infarction due to coronary thrombosis at the site of eccentric dilatation in the first case of coronary artery involvement in a patient with sJIA, as reported by Parry et al. [9]. Another patient with sJIA presenting with right coronary artery dilatation with thrombosis was reported by Shaikh et al. [10]. These case reports suggest that coronary artery dilatation in children with sJIA may require antithrombotic prophylaxis, at least until the coronary artery dilatation resolves. Lefèvre et al. [11] reported on four patients with sJIA and coronary artery abnormalities. Their patients were dependent on high-dose steroids, and after close monitoring, the coronary artery abnormalities resolved within 3 months. Moreover, all but one of these patients eventually required biological therapy. This suggests that coronary artery disease is associated with a poor prognosis in the early phase of sJIA. In a retrospective study of 50 patients with sJIA [12], 12 patients had echocardiographic findings, of which three patients with SoJIA had left anterior descending or right coronary artery z scores ≥2.5; 1 had a right coronary artery z score ≥ 3. All five patients were treated with corticosteroids early in the disease, which were subsequently tapered. Coronary artery dilation normalized within 4 months of disease onset. In this retrospective study, echocardiography may be more common in sJIA patients with clinical features suggestive of KD. This may lead to an increased likelihood of detecting coronary artery dilatation, with some bias in its incidence. However, it also suggests that children with sJIA who do not have clinical features suggestive of KD may be missed in clinical practice, resulting in coronary damage going undetected. Therefore, regular screening of cardiac function and echocardiography should be included in the routine assessment of sJIA.

**Table 2:** A summary of current sJIA-related studies on coronary artery damage

Study	Number of subjects	number of coronary artery lesions	Patient (No.)	Age at inclusion	Coronary artery damage manifestation	Treatment	Coronary recovery time
Gonca Keskindemirci	1	1	1	2 years	Left coronary artery dilatation(z score:3.23),Left anterior descending artery dilatation(z score:4.83)	Methotrexate,Steroid, Anakinra,Cyclosporin, Prednisolon	6 months
Shakeel Shaikh	1	1	1	6 years	right coronary artery dilatation(5.7 - 6.2 mm) with Thrombus formation	Methylprednisolone	2 weeks
Alain Lefèvre-Utile	29	4	1	10 months	Left coronary artery dilatation of 3 mm, a pericardial effusion	High doses of systemic steroids, MTX,Etanercept, Canakinumab,Roactemra	3 months
			2	14 months	Pericardial effusion (5,6 mm) and hyperechogenicity with irregularity of the left coronary artery	High doses of systemic steroids	1 week for the artery dilatation and 1 month for pericarditis
			3	2 years	Thickened left coronary artery walls	High doses of systemic steroids, Tocilizumab	1 week
			4	14 years	a hyperechogenicity of the left and right arteries	High doses of systemic steroids, Anakinra	2 weeks
Bryce A.Binstadt	12	5	1	4 years	Left coronary artery dilatation(z score:3.19),Left anterior descending artery dilatation(z score:2.47)	Methotrexate	4 months
			2	6 years	Right coronary artery dilatation(z score:3.18)	Methotrexate, Infliximab	5 Days
			3	2 years	Right coronary artery dilatation(z score:2.53)	Methotrexate, Naproxen	5 Days
			4	1 year	Left anterior descending artery dilatation (z score:2.2),Right coronary artery dilatation(z score:2.08)	Methotrexate, Prednisolone, Etanercept, Thalidomide, Methylprednisolone	1 month
			5	10 years	Left anterior descending artery dilatation (z score:2.18)	Methotrexate, Hydroxychloroquine	1 month
Sharath Kumar	1	1	1	6 years	Right coronary artery dilatation(2.7 mm),Left coronary artery dilatation(3.3 mm),	Methylprednisolone, Cyclosporine, Etoposide,Dexamethasone	2 weeks
Li SN	5	5	1	7 months to 4 years and 7 months	Unilateral coronary artery dilation	Methylprednisolone,Methotrexate	1-3 months
			2		Unilateral coronary artery dilation	Methylprednisolone,Methotrexate	
			3		Unilateral coronary artery dilation	Methylprednisolone, Cyclosporine,Tocilizumab,Methotrexate,	
			4		Bilateral coronary artery dilation	Methylprednisolone, Cyclosporine,Tocilizumab,Methotrexate,	
			5		Bilateral coronary artery dilation	Methylprednisolone, Cyclosporine,Tocilizumab, Methotrexate	
*A. Felix*	25	4	1	3.8 years	Coronary artery dilation(3.6 mm)	MTX,Anakinra,Roactemra, Methylprednisolone	2 months
			2	4.4 years	Coronary artery dilation(3.4 mm)	Methylprednisolone	2 months
			3	7 years	Coronary artery dilation(5.2 mm)	Methylprednisolone	2 months
			4	14.3 years	Coronary artery dilation(4.2 mm)	Anakinra, MethylpredNisolone	2 months

The prevalence of MAS in sJIA is unknown, but 10-15% of children with sJIA have MAS, and up to 40% of children with sJIA have subclinical MAS [13, 14]. Several studies have found that coronary artery damage in children with sJIA combined with MAS may be associated with a poor prognosis. A 6-year-old child diagnosed with sJIA combined with MAS [15], whose echocardiogram showed dilated and tortuous coronary arteries, was poorly treated with two courses of methylprednisolone shock therapy, and his disease eventually resolved when he was treated with cyclosporine and etoposide and dexamethasone. Li SN et al. [16] studied five children with sJIA, three with unilateral coronary artery dilatation, and two with bilateral coronary artery dilatation. Four had combined multi-organ injury, and three had combined macrophage activation syndrome. It was found that sJIA with combined coronary artery dilatation was severe and insensitive to hormones and conventional immunosuppressive agents, requiring the addition of biological therapy. Some Afro-Caribbean children with sJIA show features such as higher rates of MAS and coronary artery involvement at presentation [17]. Four out of 25 children (16%) had coronary artery involvement at presentation. More than half (52%) had macrophage activation syndrome (MAS) in childhood. Coronary artery involvement was present at diagnosis (16%); all had coronary artery dilation (from 3.4 to 5.2 mm) without aneurysms. In the cohort studied, children with MAS who had coronary artery involvement required biological therapy to control the disease. All three studies found that coronary artery dilatation was an indicator of poor prognosis in sJIA, suggesting that biologic therapy should be added as early as possible and that early treatment improves the prognosis of coronary artery dilatation.

The incidence and mechanism of sJIA-induced coronary artery injury are not fully understood, and some researchers have suggested that it may be related to altered coronary hemodynamics and endothelial damage in inflammatory states [18]. High levels of C-reactive protein (CRP) and increased pro-inflammatory cytokines (e.g., tumor necrosis factor-alpha (TNF-alpha) and interleukin (IL)-1 and interleukin-6) are commonly reported in JIA [19], perhaps in association with these inflammatory storms. The extent of their coronary involvement varies.

The mechanism of coronary artery damage in patients with sJIA combined with MAS is not fully understood, but some potential mechanisms have been suggested. In children with sJIA, the hyperinflammatory state is not controlled promptly and, under the influence of various triggers, leads to overactivation and proliferation of T lymphocytes and macrophages, resulting in a “cytokine storm,” including the overproduction of cytokines such as IL-6 and IL-18, interferon (IFN)-γ and TNF-α, etc., and the over release of these cytokines can lead to coronary inflammation and vascular endothelial injury [20]. Alternatively, in the context of immune regulation, in patients with sJIA combined with MAS, an imbalance in the regulation of the immune system may lead to an enhanced autoimmune response, which in turn may trigger coronary damage. Immune dysregulation may include abnormal activation and function of T cells and macrophages [21]. The incidence of coronary artery damage in sJIA is relatively low, and there are no definitive data or statistics available; however, some studies have shown that cardiac involvement is an essential clinical feature in patients with sJIA combined with MAS. Cardiac involvement can include myocarditis, pericarditis, and coronary artery inflammation, but the incidence of coronary artery damage remains unclear [22].

**Fig. 2:**
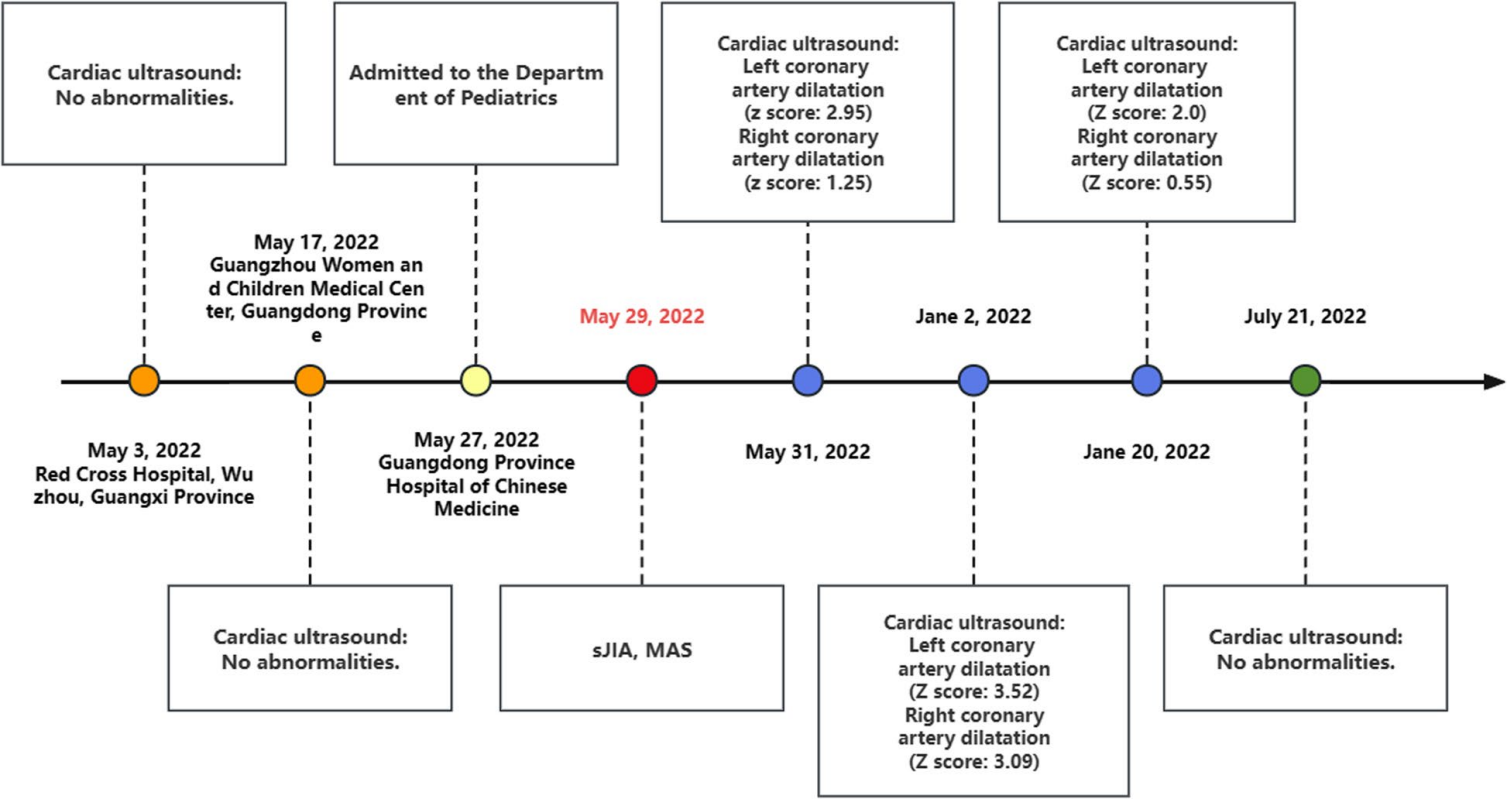
Time Distribution of Coronary Injury

The children in this study underwent two cardiac echocardiograms during their initial hospitalization, which showed no abnormalities, so they were considered to have incomplete KD and were treated with shock doses of IVIG, which was unsatisfactory. After further investigation, sJIA-MAS was considered. Although coronary artery damage is not typical in sJIA-MAS, the child’s cardiac enzymes suggested that there were abnormalities in cardiac function, so although the child’s prehospital investigations did not reveal any coronary artery abnormalities, UCG was repeated with caution, and the child was found to have abnormalities such as coronary artery dilatation and pericardial effusion 4 days after hospital admission. In this case, no cardiac abnormality was found in the early stage of the disease, but coronary artery damage was discovered after the diagnosis of sJIA-MAS.This may suggest that without timely intervention in the early stages of sJIA, coronary artery damage is more likely to occur after progression to sJIA-MAS. Specific coronary changes are shown in Fig. 2.

Coronary artery damage in sJIA is a clinical challenge, and clinicians should be aware that early diagnosis is the key to preventing disease progression and improving disease prognosis. Although abnormalities in cardiac enzymes and other related markers do not necessarily indicate coronary artery damage, a dynamic review of echocardiography is still recommended for children diagnosed with sJIA who have changes in cardiac enzymes and other related markers. The advantage of a UCG is that it can detect lesions in time and assess the severity of lesions, cardiac function, and hemodynamic changes; therefore, a dynamic review of UCG is of great importance for the early diagnosis of cardiovascular damage in sJIA and the follow-up of the disease.

## Conclusion

We report a rare case of coronary artery damage in a child with sJIA combined with MAS. Coronary artery damage in sJIA is a clinical challenge and should be taken seriously by clinicians. In clinical practice, children with sJIA who do not have the clinical features of KD may be overlooked, and coronary artery disease may go undetected. The presence of coronary artery damage may be associated with a poor prognosis and should be treated with biological agents as early as possible. Antithrombotic prophylaxis is recommended early in the presence of coronary artery disease, at least until coronary artery dilatation resolves. Long-term follow-up is necessary until the coronary arteries return to normal, especially in children with combined MAS. Further studies are also needed to identify markers of early coronary involvement for early intervention and improved prognosis. More studies are needed.

### Ethics statement

The case report did not involve any personal private information, and the collected information did not include the contact information of the patient’s family members, and it was impossible to get in touch with them. According to national legislation and institutional requirements, the written informed consent of the participant’s legal guardian/close relative was not required.

## Data Availability

The raw data for the conclusions of this paper can be provided to any qualified researcher.

## Abbreviations

SJIA: Systemic juvenile idiopathic arthritis
MAS: Macrophage activation syndrome
VIG: Intravenous immune globulin
KD: Kawasaki disease
UCG: Ultrasonic Cardiogram
